# Gender Inclusivity and Exclusivity in US Hospitals’ Online Obstetrics, Labor and Delivery, and Pregnancy-Related Resources: Cross-Sectional Study

**DOI:** 10.2196/93770

**Published:** 2026-07-22

**Authors:** Charlie O Coward, Jennifer L Howell

**Affiliations:** 1 Department of Psychological Sciences, University of California, 5200 N Lake Road, Merced, CA, 95343, United States, 1 -209-228-4723

**Keywords:** transgender, pregnancy, reproductive health, gender-inclusive language, perinatal care

## Abstract

**Background:**

Research on transgender people’s health, particularly in reproductive health, has expanded exponentially over the past decades. However, previous studies frequently highlight perceived inaccessibility and gender-exclusivity of reproductive and perinatal care for transgender people.

**Objective:**

This observational study used a cross-sectional content analysis to examine online obstetrics, labor and delivery, and pregnancy-related materials from a purposive sample of 178 online hospital resources across the United States to understand the extent to which they use gender-inclusive and gender-exclusive language, imagery, and other inclusive symbolism.

**Methods:**

Two hundred US hospitals were purposively selected from the most populous cities in each state; 22 (11%) lacking obstetrics pages were excluded, yielding 178 (89%) hospitals across all 50 states. Four coders assessed obstetrics websites (from October 2025 to December 2025) for transgender-specific resources, inclusive language, second-person language, exclusive language, inclusive imagery, and inclusive symbols or statements. Hospitals were categorized by US Census region, transgender-specific legal protections, and university or religious affiliation.

**Results:**

Across the United States, the presence of gender-inclusive elements was relatively rare, while most hospitals consistently used second-person or gender-exclusive elements. These patterns persisted across geographic regions, university affiliations, religious affiliations, and variation in state-level, transgender-specific legal protections, although hospitals in states with stronger protections and university-affiliated institutions showed somewhat higher rates of inclusive content. Importantly, even on websites that included gender-inclusive language, such content co-occurred with gender-exclusive terms, raising questions about how transgender and gender-diverse patients might interpret these mixed signals when deciding where to seek care.

**Conclusions:**

These findings underscore the need for hospitals to develop clear, service-specific standards for gender-inclusive obstetric communication and to ensure that online materials visibly and consistently signal their readiness to care for transgender and gender-diverse patients across the perinatal period.

## Introduction

### Background

Transgender and gender-diverse (TGD) people—those whose sex assigned at birth does not match their current gender identity—are increasingly choosing to build families through pregnancy [1], and scholarship over the past decade has underscored the importance of access to identity-sensitive, affirming health care across the perinatal period [2,3]. Pregnancy can heighten the visibility and salience of gender identity for many TGD people [4–6] and consequently increase exposure to discrimination, stigma, and social isolation [2,7,8]. These stressors have been linked to adverse outcomes for pregnant people, including poorer mental health [5,9] and elevated obstetric risk [10].

Health care providers may serve as a key buffer against these risks by offering identity-sensitive care in perinatal settings. Previous research in cisgender person samples has shown that positive communication and support from obstetricians throughout pregnancy are associated with better self-care and lower psychological distress [11]. However, TGD patients report medical maltreatment, including misgendering [12] and harassment from physicians [13,14], at a much higher rate than their cisgender counterparts [15]. To mitigate the likelihood of such maltreatment while seeking perinatal care, some TGD patients may extensively review available online resources and hospital materials for information or cues about policies, values, and their possible patient experience [16]. Indeed, environmental cues can signal whether a context is safe or threatening, particularly in settings involving identity-relevant evaluation or potential stigma [16–18]. In health care contexts, even subtle signals—including language, imagery, and visible nondiscrimination statements—can function as a “safety cue” that has been shown to shape expectations of respect, belonging, and risk [19].

Although previous research and guidelines increasingly recommend gender-inclusive communication and materials in perinatal settings [20,21], systematic data on how US hospitals present pregnancy-related care online, particularly with respect to TGD inclusivity, remain limited. Indeed, previous reviews of health system websites have been constrained to specific locations (eg, New York, New Jersey, and Connecticut [22]) and have generally focused on narrow subsets of institutions, leaving national patterns of online obstetric gender diversity and inclusivity largely unexamined. Additionally, previous studies have focused only on the presence of gender-inclusive language and symbols rather than examining gender-exclusive or gender-neutral language that appears in the same spaces. Thus, the present study aimed to examine obstetric-related website content from large hospitals across the United States to understand how gender-inclusive (vs gender-exclusive) online content varies across geographic regions and whether it relates to state-level transgender-specific legal protections. Specifically, we had two primary research questions:

1. Gender-inclusive, gender-neutral, and gender-exclusive language and symbols: How frequently do hospital websites use gender-exclusive language (eg, “women”), gender-neutral language (eg, “you”), or gender-inclusive language (eg, “pregnant people”) and/or specific LGBTQ+ (lesbian, gay, bisexual, transgender, queer or questioning, plus [others])-inclusive imagery, statements, or symbols?
2. Moderating factors: Are hospitals located in states with more progressive policy environments—specifically, those with explicit transgender-specific legal protections—more likely to use inclusive language, imagery, and statements than hospitals in less protective states? Does the use of inclusive language differ according to whether a hospital is public or private and whether it is religiously affiliated or not?

### Summary of the Study Aim

The present study aimed to examine the prevalence of gender-inclusive and gender-exclusive language in the online content of large hospitals’ obstetric-related websites.

## Methods

### Design: Hospital Website Selection

Two hundred hospitals were initially selected using criteria designed to capture institutions that are both influential within health systems and likely to serve large, diverse patient populations while remaining feasible for the website-review team. First, the sampling frame included “flagship” institutions identified through the *2025 US News & World Report* rankings, which highlight hospitals recognized for clinical excellence and broad reach (eg, Johns Hopkins, Cleveland Clinic, and Mayo Clinic [23]). Such organizations may shape communication norms and online practices adopted by other facilities [24]. Second, to approximate patient volume and website user frequency, we chose hospitals located in or near the most populous city in each US state, using city population as a proxy for service volume and web traffic [22,25]. After selection, 22 hospitals were excluded from coding because they did not have any part of their web page dedicated to obstetrics. The final analytic sample included 178 hospitals distributed across all 50 US states. The 22 excluded hospitals did not differ significantly from the sample in their representation across geographic regions, state-level transgender protections, public vs private status, university affiliation, or religious affiliation [26]. A final list of included hospitals appears in Table S1 in Multimedia Appendix 1. Websites were accessed and coded from October 2025 through December 2025.

### Coding Procedure

#### Region, Transgender Protections, and State Equality Index

Using their state location, we assigned each hospital to a broader geographic region (ie, Northeast, Midwest, South, or West). We also assigned each hospital to a state-level transgender legal protection category based on the *Human Rights Campaign* (HRC) index, which identifies whether states have explicit legal protections for transgender people (yes or no; accessed December 2025 [27]). Finally, we assigned each hospital a state-based transgender health care score based on the Movement Advancement Project’s Gender Identity Healthcare score [28]. A list of each hospital with its region, transgender protection category, and transgender health care score appears in Table S1 in Multimedia Appendix 1.

#### Content Inclusivity

Four independent coders reviewed the hospital’s official website, focusing on obstetrics, labor and delivery, and pregnancy-related content. For each hospital, coders evaluated the presence or absence of the following features:

1. Transgender-specific pregnancy resources: coders noted whether at least one page or section explicitly acknowledged or addressed TGD patients in the context of pregnancy, such as information about gender-affirming care during conception, prenatal care, delivery, or postpartum care.
2. Inclusive language: coders identified whether the website used explicitly gender-inclusive terminology to refer to the gestating person, including terms such as “pregnant people,” “parent,” or “chestfeeding” in lieu of exclusively gendered terms such as “pregnant women,” “mother,” or “breastfeeding.”
3. Second-person language: coders recorded whether the website used second-person language (eg, “your pregnancy” and “your baby”) as a form of address that does not specify gender.
4. Exclusive language: coders noted instances in which the website used gender-exclusive terminology (eg, “pregnant women” and “mothers”) in contexts where a gender-inclusive alternative could have reasonably been used. Department and specialty names that reflect institutional or clinical conventions (eg, “Women’s Health” and “Maternal-Fetal Medicine”) or descriptions in which the gender-exclusive term accurately reflected the population (eg, describing studies that included only pregnant women) were not coded as exclusive.
5. Gender-inclusive imagery: coders assessed whether the website included imagery that visually represented gender or sexual diversity in perinatal contexts, such as pregnant people who were not feminine-presenting or parenting couples who were not heterosexual-presenting.
6. Inclusive symbols, phrases, or statements: coders documented diversity, equity, and inclusion (DEI) statements, pride flags, LGBTQ+-affirming iconography, or explicit references to nondiscrimination and inclusion policies relevant to LGBTQ+ patients.

Interrater agreement was assessed independently by 2 coders for each website. The coders agreed on 97.7% of all codes. In the remaining 2.3% of cases of disagreement, a third coder reviewed the relevant web pages, and the coders agreed on a final coding decision. After all coding was finalized, the lead author rereviewed 10% of the data for each region and found only a single point of coding disagreement, which was resolved with the original coders.

### Ethical Considerations

Because this was an observational study of publicly available websites, it did not require institutional review board approval or formal consent.

## Results

### Patterns of Inclusivity

Table 1 displays the coding results. Across the full sample, explicitly transgender-inclusive resources and gender-inclusive imagery (11/178, 7%) remained rare [1]. Although there was overlap, the hospitals that included transgender-inclusive resources and those that included gender-inclusive imagery were not the same. Almost all hospitals used at least one instance of gender-exclusive language (n=173, 98%; eg, “mother,” “women,” or “maternity”) in contexts in which more gender-neutral alternatives would have been possible. At the same time, nearly all hospitals used at least one instance of second-person, gender-neutral phrasing (n=174, 98%; eg, “your pregnancy,” or “your baby”), suggesting that neutral language practices already coexist alongside gendered terminology within many institutions.

**Table 1.** Content coding of indicators of gender inclusivity in hospital obstetrics websites.

Characteristics	Transgender-specific resources (n=10), n (%)	Gender-inclusive language (n=50), n (%)	Second-person language (n=174), n (%)	Gender-exclusive language (n=173), n (%)	Gender-inclusive imagery (n=11), n (%)	Inclusive symbols or phrases (n=92), n (%)
By region						
Northeast	4 (10.8)	15 (40.5)	37 (100)	35 (94.6)	4 (10.8)	20 (54.1)
Midwest	4 (9.1)	12 (27.3)	43 (97.7)	44 (100)	5 (11.4)	27 (61.4)
South	1 (2.2)	12 (26.7)	45 (100)	45 (100)	0 (0)	19 (42.2)
West	1 (1.9)	11 (21.2)	49 (94.2)	49 (94.2)	2 (3.8)	26 (50)
By state-level legal protection status						
Legal protections	9 (8.9)	31 (30.7)	96 (95)	102 (97.1)	10 (9.9)	55 (54.4)
No legal protections	1 (1.4)	19 (26.4)	72 (100)	75 (100)	1 (1.4)	35 (48.6)
By university affiliation						
University affiliated	9 (10.5)	29 (33.7)	84 (97.7)	82 (95.3)	10 (11.6)	52 (60.5)
Not university affiliated	1 (1.1)	21 (22.8)	90 (97.8)	91 (98.9)	1 (1.1)	40 (43.5)
By religious affiliation						
Religiously affiliated	0 (0)	7 (17.1)	39 (95.1)	40 (97.6)	0 (0)	14 (34.1)
Secular	10 (7.3)	43 (31.4)	135 (98.5)	133 (97.1)	11 (8)	78 (56.9)

Despite the limited availability of transgender-specific content, there were notable signals of broader LGBTQ+ support. Nearly half of the hospitals included some form of DEI statement or LGBTQ+ iconography, such as pride flags or explicit references to caring for LGBTQ+ patients (92/178, 52%). Additionally, many hospitals featured at least one instance of gender-inclusive language (n=50, 28%; eg, “pregnant people”) on their obstetrics pages, indicating that some institutions have begun to incorporate more affirming terminology into pregnancy materials.

### Regional Variation in Inclusivity Patterns

We conducted a series of Fisher-Freeman-Halton (FFH) exact tests to examine whether the outcomes differed by region in a 2 (outcome: yes or no)×4 (region: Northeast, Midwest, South, or West) contingency table. There was a significant regional difference in gender-inclusive imagery (FFH *χ*^2_3_^=7.1; *P*=.05; Cramer *V*=0.20). Specifically, most hospitals with gender-inclusive imagery (9/12, 75%) were located in either the Northeast (n=4, 33% of all hospitals with gender-inclusive imagery) or the Midwest (n=5, 42% of all hospitals with gender-inclusive imagery). Indeed, none of the hospitals in the South and only 3 (6%) of the 52 hospitals in the West included gender-inclusive imagery.

There were no statistically significant regional differences in transgender-specific pregnancy resources (FFH χ^2_3_^=4.9; *P*=.16; Cramer *V*=0.17), gender-inclusive language (FFH *χ*^2_3_^=4.01; *P*=.26; Cramer *V*=0.15), second-person language (FFH *χ*^2_3_^=3.4; *P*=.25; Cramer V=0.17), gender-exclusive language (FFH *χ*^2_3_^=4.4; *P*=.12; Cramer V=0.17), or inclusive symbols or phrases (FFH *χ*^2_3_^=3.4; *P*=.34; Cramer V=0.14). Although the overall regional difference was not statistically significant, gender-inclusive language was 1.6 times more common among hospitals in the Northeast (41%) than among hospitals in other regions (mean 25%, SD 0.43%; Northeast vs other regions: *χ*^2_1_^=3.6; *P=*.06; φ=0.14).

### State-Level Legal Protections and Equality

We conducted a series of Fisher exact tests to examine whether the outcomes differed as a function of state-level protections in a 2 (outcome: yes or no)×2 (legal protections: yes or no) contingency table. There were significant differences in both transgender-specific pregnancy resources (*χ*^2_3_^=4.4; exact *P=*.047; φ=0.16) and gender-inclusive imagery (*χ*^2_3_^=5.1, exact *P=*.03; φ=0.17) as a function of transgender-specific legal protection status. Among hospitals in states without such protections (72/178, 40%), only 1 hospital included transgender-specific pregnancy resources, and only 1 hospital featured gender-inclusive imagery. By contrast, in states with such protections (n=101, 57%), 9 hospitals included transgender-specific resources, and 10 included gender-inclusive imagery. There was also a marginally significant difference in gender-exclusive language as a function of transgender-specific legal protection status (*χ*^2_3_^=3.7; exact *P=*.08; φ=0.15). Specifically, although every hospital in states without legal protections used gender-exclusive language, 5% of hospitals in states with legal protections did not use gender-exclusive language. There were no significant differences as a function of transgender-specific legal protection status in gender-inclusive language (*χ*^2_3_^=0.4; exact *P=*.51; φ=.05), second-person language (*χ*^2_3_^=0.5; exact *P=*.64; φ=–0.05), or inclusive symbols or phrases (*χ*^2_3_^=0.6; exact *P=*.54; φ=0.06).

We conducted a series of point-biserial correlations to examine the relationship between a hospital’s state-level transgender health care score and the inclusiveness of its website. There was a significant positive correlation with transgender-specific pregnancy resources (*r*_176_=0.18; *P*=.02) and gender-inclusive imagery (*r*_176_=0.18; *P*=.02), such that hospitals in states with better transgender health care scores were more likely to have transgender-specific resources and use gender-inclusive imagery. There was also a marginal negative correlation with gender-exclusive language (*r*_176_=−0.14; *P*=.06), such that hospitals in states with higher transgender health care scores were less likely to use gender-exclusive language. A hospital’s state transgender health care index did not predict gender-inclusive language use (*r*_176_=0.07; *P*=.33), second-person language use (*r*_176_=−0.01; *P*=.86), or the use of inclusive symbols or phrases (*r*_176_=0.11; *P*=.14).

### University Affiliation and Inclusivity

We conducted a series of Fisher exact tests to examine whether the outcomes differed as a function of a hospital’s university affiliation in a 2 (outcome: yes or no)×2 (university affiliation: yes or no) contingency table. There were significant differences in transgender-specific pregnancy resources (*χ*^2_3_^=7.4; exact *P=*.008; φ=0.20), gender-inclusive imagery (*χ*^2_3_^=8.5; exact *P=*.004; φ=0.22) and inclusive symbols and phrases (*χ*^2_3_^=5.1; exact *P=*.03; φ=0.17), as a function of university affiliation. Specifically, compared to hospitals without a university affiliation (92/178, 52%), university-affiliated hospitals (86/178, 48%) more frequently offered transgender-specific pregnancy resources (9/86, 10% vs 1/92, 1.1%), had gender-inclusive imagery (10/86, 12% vs 1/92, 1.1%), and had inclusive symbols or phrases (52/86, 60% vs 40/92, 43%). There were no significant differences as a function of university affiliation in gender-inclusive language (*χ*^2_3_^=2.6; exact *P=*.13; φ=0.12), second-person language (*χ*^2_3_^=0.005; exact *P*≥.99; φ=−0.005), or gender-exclusive language (*χ*^2_3_^=2.1; exact *P=*.19; φ=−0.11).

### Religious Affiliation and Inclusivity

We conducted a series of Fisher exact tests to examine whether the outcomes differed as a function of religious affiliation in a 2 (outcome: yes or no) × 2 (religious affiliation: yes or no) contingency table. There was a significant difference in inclusive symbols and phrases as a function of religious affiliation (*χ*^2_3_^=6.6; exact *P=*.01; φ=−0.19). Religiously affiliated hospitals (41/178, 23%) had gender-inclusive symbols or phrases (34%) less frequently than did secular hospitals (137/178, 77% hospitals; 57% with inclusive symbols or phrases). There were marginally significant differences in gender-inclusive language (*χ*^2_3_^=3.2; exact *P=*.08; φ=−0.13) and gender-inclusive imagery (*χ*^2_3_^=3.5; exact *P=*.07; φ=−0.14). Religiously affiliated hospitals used gender-inclusive language (7/41, 17%) less frequently than secular hospitals (42/137, 31%), and none of the religiously affiliated hospitals had gender-inclusive imagery, compared to 8% (11/137) of secular hospitals. There were no significant differences as a function of religious affiliation in transgender-specific pregnancy resources (*χ*^2_3_^=3.2; exact *P=*.12; φ=−0.13)—though notably, no religiously affiliated institution offered such resources—second-person language (*χ*^2_3_^=1.7; exact *P=*.23; φ=−0.10) or gender-exclusive language (*χ*^2_3_^=0.03; exact *P*≥.99; φ=0.01).

## Discussion

### Principal Findings

Across a large, geographically diverse sample of hospitals, gender-inclusive resources, terminology, and imagery on obstetrics websites were relatively rare, even in states with transgender-specific legal protections and among nonreligious and university hospitals. These findings align with qualitative reports from TGD people who describe pregnancy and birth care as structured around cisgender women’s experiences, leading to feelings of both erasure and hyper-visibility in clinical spaces [4,29]. Although hospitals in more protective states—particularly those in the Northeast—showed somewhat higher rates of gender inclusivity and transgender-specific pregnancy resources, inclusive language remained low everywhere, whereas gender-exclusive language was nearly ubiquitous. This pattern suggests a pervasive baseline of cisnormative presentation of pregnancy across US hospital websites, in which transgender-specific needs are still largely invisible, regardless of environment.

The near-universal use of second-person language raises important questions about how TGD patients interpret gender-neutral phrasing, especially when it is embedded within predominantly gendered content. Although it may be tempting to assume that any instance of gender-inclusive language, including second-person language, is inherently positive, its impact might depend on how it is integrated into the broader message framework. Previous research has noted that additive inclusion—in which inclusive language is appended to references to cisgender women (eg, “mothers and other pregnant people”)—may be experienced as othering and may signal to TGD patients that services remain primarily oriented toward cisgender women, while TGD patients are positioned as secondary [21]. At present, however, there is little empirical research that directly examines how TGD patients interpret second-person obstetric language as inclusive vs exclusive, particularly when it co-occurs with both gender-inclusive and gender-exclusive terms.

Similarly, the frequent coexistence of gender-exclusive obstetrics language with DEI statements and LGBTQ+ iconography suggests a disconnect between institutional branding and the specific information available to patients seeking pregnancy care. Prior work on transgender health web pages and TGD reproductive care cautions that generic statements of inclusion without concrete descriptions of identity-sensitive care may be interpreted as a form of symbolic inclusion, which may inadvertently erode trust between patients and health care systems [20]. This misalignment highlights the need for research that specifically tests how TGD patients interpret combinations of symbolic cues and service-specific information and suggests that institutions should make a greater effort to align online obstetric content with the inclusive values they endorse.

Although no hospital in the present sample consistently demonstrated the comprehensive use of gender-inclusive language and affirming symbols across its obstetric-related websites, it is important to note that such practices are feasible. For example, organizations such as Planned Parenthood—although not included in the current sample because they comprise a series of clinics rather than a large hospital system—have developed online materials that consistently use gender-neutral language (eg, “your baby” and “most people”). Additionally, in their gender-affirming care section, they explicitly acknowledge TGD people as capable of pregnancy and that their health centers are “open to all genders.” These examples suggest that inclusivity does not need to be highly visible or represent a comprehensive redesign of all patient-facing materials; even subtle choices, such as avoiding exclusively gendered terms such as “women,” may function as meaningful cues that reduce anticipated exclusion among TGD patients. However, online inclusivity is only one aspect of a broader institutional commitment to affirming care—meaningful change also requires obstetrician and staff training, as well as the consistent implementation of affirming practices in clinical interactions.

### Limitations

The present study extends prior work on online representations of TGD health by focusing specifically on obstetric websites across a large, geographically diverse hospital sample. However, there are some limitations to consider. This study relied on a purposive sample of prominent hospitals within the most populous city in each state, which tend to adopt more progressive social policies than rural settings in the same state [30]. Consequently, the findings may overestimate the prevalence of gender-inclusive content. Future work would benefit from stratified sampling across urban and rural hospitals to evaluate whether observed patterns vary by rurality.

All websites were reviewed between October and December 2025, during a period when US federal policies and public discourse around gender-affirming care and transgender rights were negative in tone and supported transgender person exclusion [31]. It is possible that some hospitals that had previously adopted or considered adopting more explicitly gender-inclusive language or imagery subsequently revised or removed this content in response to political, legal, or institutional pressures. Future studies might repeat this review either internationally or under revised US policy climates.

### Conclusions

Taken together, these findings highlight an opportunity for hospitals to move beyond generic DEI branding and develop service-specific standards for gender-inclusive content. Professional societies and hospital systems offer concrete guidance on language, imagery, and resource provision in perinatal settings, including recommendations for explicitly naming TGD patients, describing available gender-affirming pregnancy care, and ensuring that nondiscrimination policies and identity safety cues are easily visible on obstetric pages [20,32,33]. For example, hospitals could ensure that online obstetric materials, at a minimum, use second-person, gender-neutral language and acknowledge noncisgender gestating people. Such changes may help reduce anticipatory stigma and signal that obstetric services are prepared to care for TGD patients across the perinatal period.

## Supplementary material

10.2196/93770 Multimedia Appendix 1 Hospitals and transgender-specific state protections.

## Abbreviations

DEI: Diversity, equity, and inclusion
FFH: Fisher-Freeman-Halton
HRC: Human Rights Campaign
LGBTQ+: lesbian, gay, bisexual, transgender, queer or questioning, plus (others)
TGD: transgender and gender-diverse
