# Supplementary material for: Gender Inclusivity and Exclusivity in US Hospitals’ Online Obstetrics, Labor and Delivery, and Pregnancy-Related Resources: Cross-Sectional Study
Source: J Med Internet Res. 2026 Jul 22;28:e93770. doi: 10.2196/93770 (PMC13392649; doi:10.2196/93770)
Supplement: Multimedia Appendix 1 [file jmir-v28-e93770-s001.docx]

Table 1: Final List of Hospitals

| **Hospital** | **State** | **Trans-specific protections** |
| --- | --- | --- |
| **Region: Midwest** | | |
| - MercyOne Des Moines Medical Center | IA | No |
| - UnityPoint Health-Iowa Methodist Medical Center | IA | No |
| - Advocate Christ Medical Center | IL | Yes |
| - Endeavor Health NorthShore Hospitals | IL | Yes |
| - Loyola University Medical Center | IL | Yes |
| - Northwestern Medicine - Northwestern Memorial Hospital | IL | Yes |
| - Rush University Medical Center | IL | Yes |
| - Silver Cross Hospital | IL | Yes |
| - University of Chicago Medical Center | IL | Yes |
| - Community Hospital (East, North, Anderson & Madison County, South) | IN | No |
| - Eskenazi Health-Indianapolis | IN | No |
| - Fanciscan Health Indianapolis | IN | No |
| - Indiana University Health Medical Center | IN | No |
| - NMC Health-Newton | KS | Yes |
| - Wesley Healthcare Center | KS | Yes |
| - Norton Hospitals | KY | No |
| - University of Louisville Hospital | KY | No |
| - Corewell Health William Beaumont University Hospital | MI | Yes |
| - DMC Detroit | MI | Yes |
| - Henry Ford Hospital | MI | Yes |
| - Allina Health | MN | Yes |
| - Hennepin Healthcare | MN | Yes |
| - University of Minnesota Medical Center | MN | Yes |
| - North Kansas City Hospital | MO | No |
| - Research Medical Center | MO | No |
| - Saint Luke's Hospital of Kansas City | MO | No |
| - University Health | MO | No |
| - Essentia Health-Fargo | ND | No |
| - CHI Health | NE | No |
| - Creighton University Medical Center | NE | No |
| - Nebraska Medicine | NE | No |
| - Nebraska Methodist Hospital | NE | No |
| - Cleveland Clinic | OH | No |
| - Mount Carmel East Hospital | OH | No |
| - Ohio State University Wexner Medical Center | OH | No |
| - OhioHealth | OH | No |
| - AHN West Penn Hospital | PA | Yes |
| - Hospitals of the University of Pennsylvania-Penn Presbyterian | PA | Yes |
| - Pennsylvania Hospital | PA | Yes |
| - UPMC Magee-Women's Hospital | PA | Yes |
| - Sanford USD Medical Center | SD | No |
| - Aurora St. Luke's Medical Center | WI | Yes |
| - Froedtert Hospital and the Medical College of Wisconsin | WI | Yes |
| - University of Wisconsin University Hospital | WI | Yes |
| **Region: Northeast** | | |
| - Bridgeport Hospital | CT | Yes |
| - Danbury Hospital | CT | Yes |
| - Greenwich Hospital | CT | Yes |
| - Norwalk Hospital | CT | Yes |
| - Stamford Hospital | CT | Yes |
| - Yale New Haven | CT | Yes |
| - George Washington University Hospital | DC | N/A |
| - MedStar Washington Hospital Center | DC | N/A |
| - St. Francis Hospital-Wilmington | DE | Yes |
| - Beth Israel Deaconess Medical Center | MA | Yes |
| - Boston Medical Center | MA | Yes |
| - Brigham and Women's Hospital | MA | Yes |
| - Massachusetts General Hospital | MA | Yes |
| - Tufts Medical Center | MA | Yes |
| - Greater Baltimore Medical Center | MD | Yes |
| - Johns Hopkins | MD | Yes |
| - MedStar Union Memorial Hospital | MD | Yes |
| - University of Maryland | MD | Yes |
| - Maine Medical Center | ME | Yes |
| - Northern Light Mercy Hospital | ME | Yes |
| - Catholic Medical Center | NH | No |
| - Elliot Hospital | NH | No |
| - Jersey Shore University Medical Center at Hackensack Meridian Health | NJ | Yes |
| - Newark Beth Israel Medical Center | NJ | Yes |
| - University Hospital Newark | NJ | Yes |
| - Brookdale Hospital Medical Center | NY | Yes |
| - Lenox Hill Hospital at Northwell Health | NY | Yes |
| - Long Island Jewish Medical Center | NY | Yes |
| - New York University Langone Hospitals | NY | Yes |
| - New York-Presbyterian Hospital | NY | Yes |
| - NYC Health and Hospitals | NY | Yes |
| - NYU Langone Hospitals | NY | Yes |
| - Temple University Hospital | PA | Yes |
| - Thomas Jefferson University Hospitals | PA | Yes |
| - University of Pennsylvania Hospitals | PA | Yes |
| - Women and Infants Hospital of Rhode Island | RI | Yes |
| - University of Vermont Medical Center | VT | Yes |
| **Region: South** | | |
| - Crestwood Medical Center | AL | No |
| - Huntsville Hospital | AL | No |
| - University of Alabama Birmingham | AL | No |
| - CHI St. Vincent Infirmary | AR | No |
| - UAMS Medical Center | AR | No |
| - AdventHealth Orlando | FL | No |
| - Ascension St. Vincent's Riverside Hospital | FL | No |
| - Flagler Hospital | FL | No |
| - Tampa General Hospital | FL | No |
| - UF Health Shands Hospital | FL | No |
| - Emory University Hospital | GA | No |
| - Northside Hospital Atlanta | GA | No |
| - Piedmont Atlanta Hospital | GA | No |
| - University of Florida Health Jacksonville | GA | No |
| - WellStar Kennestone Hospital | GA | No |
| - University of Kentucky Albert B. Chandler Hospital | KY | No |
| - New Orleans East Hospital | LA | No |
| - Ochsner Medical Center | LA | No |
| - Touro Infirmary | LA | No |
| - Merit Health | MS | No |
| - Mississippi Baptist Medical Center | MS | No |
| - St. Dominic-Jackson Memorial Hospital | MS | No |
| - University of Mississippi Medical Center | MS | No |
| - Atrium Health Carolinas Medical Center | NC | No |
| - Duke University Hospital | NC | No |
| - Novant Health Presbyterian Medical Center | NC | No |
| - Integris Baptist Medical Center | OK | No |
| - Lakeside Women's Hospital | OK | No |
| - Mercy Hospital Oklahoma City | OK | No |
| - Oklahoma University Medical Center | OK | No |
| - Bon Secours St. Francis Hospital | SC | No |
| - MUSC Health-University Medical Center | SC | No |
| - Roper Hospital | SC | No |
| - Trident Medical Center | SC | No |
| - Nashville General Hospital | TN | No |
| - TriStar Centennial Medical Center | TN | No |
| - Vanderbilt University Medical Center | TN | No |
| - Baylor St. Luke's Medical Center | TX | No |
| - Baylor University Medical Center | TX | No |
| - Houston Methodist Hospital | TX | No |
| - Memorial Hermann Hospitals | TX | No |
| - Inova Fairfax Hospital | VA | Yes |
| - Sentara Princess Anne Hospital | VA | Yes |
| - Sentara Virginia Beach General Hospital | VA | Yes |
| - Charleston Area Medical Center | WV | No |
| **Region: West** | | |
| - Alaska Native Medical Center | AK | Yes |
| - Alaska Regional Hospital | AK | Yes |
| - U.S. Air Force Regional Hospital-Elmendorf AFB | AK | Yes |
| - Banner Boswell Medical Center | AZ | Yes |
| - Banner-University Medical Center Phoenix | AZ | Yes |
| - Chandler Regional Medical Center | AZ | Yes |
| - HonorHealth Scottsdale Shea Medical Center | AZ | Yes |
| - St. Joseph's Hospital and Medical Center | AZ | Yes |
| - Adventist Health Glendale | CA | Yes |
| - Cedars-Sinai Medical Center | CA | Yes |
| - Hoag Memorial Hospital Presbyterian | CA | Yes |
| - Huntington Health Medical Center | CA | Yes |
| - John Muir Health-Walnut Creek Medical Center | CA | Yes |
| - Kaiser Permanente Anaheim and Irvine Medical Centers | CA | Yes |
| - Keck Medical Center of USC | CA | Yes |
| - Loma Linda University Medical Center | CA | Yes |
| - MemorialCare Long Beach Medical Center | CA | Yes |
| - Scripps La Jolla Hospitals | CA | Yes |
| - Stanford Health Care-Stanford Hospital | CA | Yes |
| - Sutter Medical Center-Sacramento | CA | Yes |
| - Torrance Memorial Medical Center | CA | Yes |
| - University of California Los Angeles Medical | CA | Yes |
| - University of California San Diego Health | CA | Yes |
| - University of California San Francisco Medical Center | CA | Yes |
| - Boulder Community Health-Foothills Hospital | CO | Yes |
| - HCA HealthONE Presbyterian St. Luke's | CO | Yes |
| - Intermountain Health Saint Joseph Hospital | CO | Yes |
| - Medical Center of Aurora | CO | Yes |
| - Sky Ridge Medical Center | CO | Yes |
| - UC Health University of Colorado Hospital | CO | Yes |
| - Kapiolani Medical Center for Women and Children | HI | Yes |
| - Queen's Medical Center | HI | Yes |
| - Straub Medical Center | HI | Yes |
| - St. Alphonsus Regional Medical Center | ID | No |
| - St. Luke's Boise Medical Center | ID | No |
| - Billings Clinic | MT | No |
| - Intermountain Health St. Vincent Regional Hospital | MT | No |
| - Lovelace Medical Center | NM | Yes |
| - Presbyterian Hospital | NM | Yes |
| - University of New Mexico Hospitals | NM | Yes |
| - Centennial Hills Hospital Medical Center | NV | Yes |
| - MountainView Hospital | NV | Yes |
| - Southern Hills Hospital and Medical Center | NV | Yes |
| - Spring Valley Hospital Medical Center | NV | Yes |
| - Legacy Emanuel Medical Center | OR | Yes |
| - OHSU Hospital | OR | Yes |
| - Providence St. Vincent Medical Center | OR | Yes |
| - St. Mark's Hospital | UT | No |
| - University of Utah Hospital | UT | No |
| - Swedish First Hill Hospital | WA | Yes |
| - University of Washington Medical Center | WA | Yes |
| - Cheyenne Regional Medical Center | WY | No |
